# External Validation of SpineNet, an Open-Source Deep Learning Model for Grading Lumbar Disk Degeneration MRI Features, Using the Northern Finland Birth Cohort 1966

**DOI:** 10.1097/BRS.0000000000004572

**Published:** 2022-12-30

**Authors:** Terence P. McSweeney, Aleksei Tiulpin, Simo Saarakkala, Jaakko Niinimäki, Rhydian Windsor, Amir Jamaludin, Timor Kadir, Jaro Karppinen, Juhani Määttä

**Affiliations:** a Research Unit of Health Sciences and Technology, University of Oulu; b Finnish Institute of Occupational Health; c Department of Diagnostic Radiology, Oulu University Hospital, Oulu, Finland; d Department of Engineering Science, University of Oxford, UK; e Plexalis Ltd, Oxford, UK; f Rehabilitation Services of South Karelia Social and Health Care District, Lappeenranta; g Medical Research Center Oulu, Oulu University Hospital and University of Oulu, Finland

**Keywords:** deep learning, disk degeneration, Modic changes, SpineNet, low back pain

## Abstract

This is a retrospective observational study to externally validate a deep learning image classification model.

**Objective.:**

Deep learning models such as SpineNet offer the possibility of automating the process of disk degeneration (DD) classification from magnetic resonance imaging (MRI). External validation is an essential step to their development. The aim of this study was to externally validate SpineNet predictions for DD using Pfirrmann classification and Modic changes (MCs) on data from the Northern Finland Birth Cohort 1966 (NFBC1966).

**Summary of Data.:**

We validated SpineNet using data from 1331 NFBC1966 participants for whom both lumbar spine MRI data and consensus DD gradings were available.

**Materials and Methods.:**

SpineNet returned Pfirrmann grade and MC presence from T2-weighted sagittal lumbar MRI sequences from NFBC1966, a data set geographically and temporally separated from its training data set. A range of agreement and reliability metrics were used to compare predictions with expert radiologists. Subsets of data that match SpineNet training data more closely were also tested.

**Results.:**

Balanced accuracy for DD was 78% (77%–79%) and for MC 86% (85%–86%). Interrater reliability for Pfirrmann grading was Lin concordance correlation coefficient=0.86 (0.85–0.87) and Cohen κ=0.68 (0.67–0.69). In a low back pain subset, these reliability metrics remained largely unchanged. In total, 20.83% of disks were rated differently by SpineNet compared with the human raters, but only 0.85% of disks had a grade difference >1. Interrater reliability for MC detection was κ=0.74 (0.72–0.75). In the low back pain subset, this metric was almost unchanged at κ=0.76 (0.73–0.79).

**Conclusions.:**

In this study, SpineNet has been benchmarked against expert human raters in the research setting. It has matched human reliability and demonstrates robust performance despite the multiple challenges facing model generalizability.

Low back pain (LBP) is a major cause of disability and is estimated to be among the top 10 diseases accounting for the most disability-adjusted life years worldwide.^1^ The contribution of macroscopic structural changes in the spine to the disease burden is unclear, but some disk degeneration (DD) imaging phenotypes have been associated with LBP.^2^,^3^ Studies investigating this association are undermined by the inconsistent and subjective nature of existing grading tools. For example, Pfirrmann defines 5 grades of DD, which are assigned based on the assessment of the mid-sagittal slice of a lumbar spine T2-weighted magnetic resonance imaging (MRI) sequence.^4^ The features informing this grading scheme are subjective and its ordinal nature does not capture the continuum of DD. MRI raters’ training, experience, and individual biases add further inconsistency to the application of this commonly used classification.^5^

The automated interpretation of lumbar spine MRI using deep learning (DL) could help to overcome these reliability issues and lead to new DD imaging phenotypes that are more objective and standardized.^6^ *En masse* evaluation of large MRI data sets, which cannot feasibly be achieved using expert radiologists without significant costs, would facilitate data-driven approaches to studying DD etiology. In recent years, models have emerged that are trained to grade DD according to Pfirrmann classification and other related features such as disk height, Modic changes (MCs), and spinal stenosis.^6^–^8^ The development of these models necessitates thorough internal validation and testing as standard; however, external validation is not frequently performed.^9^,^10^ In addition, conventional machine learning metrics such as accuracy are not always helpful in the clinical setting, and internal validation tends to overestimate the true clinical performance.^9^ Without demonstrated performance on imaging data sets that are temporally and geographically distinct from those on which models have been trained, DL tools will not be applicable in either the research or clinical setting.

The work of Windsor *et al*,^11^ which is the subject of this external validation study, was among the first in the domain to report performance on a par with an expert human rater for multiple lumbar degenerative MRI features. The aim of this study was to externally validate SpineNet predictions for DD, using Pfirrmann classification and MC on data from the Northern Finland Birth Cohort 1966 (NFBC1966) (Figure 1).

**Figure 1:**
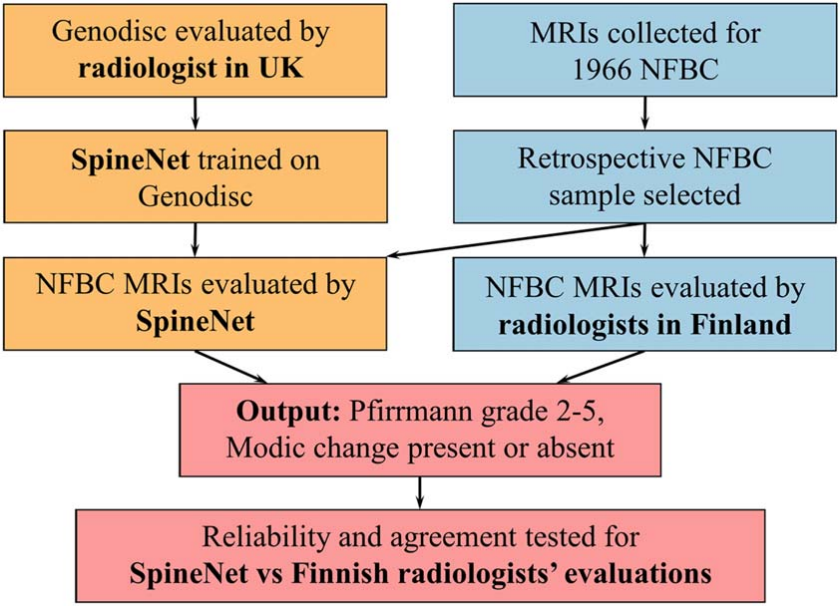
Outline of steps in the external validation process used in this study. MRI indicates magnetic resonance imaging; NFBC, Northern Finland Birth Cohort 1966.

## MATERIALS AND METHODS

### Data

The study population consists of NFBC1966, which is a prospective birth cohort started in 1965.^12^ In this study, follow-up data and MRI scans at the age of 46 years were utilized.^13^ This retrospective sample consisted of participants who underwent MRI scans between the years of 2012 and 2014 and for whom consensus DD evaluations had been carried out (n=1331). The sample size was the maximum available considering these criteria. Ethics approval for the NFBC1966 was granted by Northern Ostrobothnia Hospital District Ethical Committee. Participant information is shown in Table 1. Further information regarding the recruitment and population characteristics of this cohort, and the MRI acquisition protocols they underwent, has been described previously.^13^,^14^

**TABLE 1:** Characteristics of the Study Population

Variable	N/mean (SD)
Sex, n (%)	
Female	706 (53.0)
Male	625 (47.0)
Body mass index (kg/m^2^)	
<25	537/22.7 (1.64)
25–30	513/27.3 (1.42)
>30	280/33.7 (3.68)
LBP during last 12 mo, n (%)	
No	561 (42.0)
1–7 d	206 (15.5)
8–30 d	245 (18.4)
Over 30 d but not daily	225 (16.9)
Daily	94 (7.1)

Blinded Pfirrmann grading was carried out by two experienced musculoskeletal radiologists and one physician experienced in spinal imaging (combined experience of over 53 yr). Interrater reliability ranged from fair to good (κ=0.39–0.79). A fourth expert rater determined the consensus Pfirrmann grade.^15^ For historical reasons, Pfirrmann grades 1 and 2 were considered as grade 2. MC were evaluated by two blinded raters with specific experience in MC evaluation, with an interrater reliability of κ=0.82. MC were noted as either present or absent either side of each lumbar intervertebral disk and height and width of changes were recorded after the method outlined by Määttä *et al*.^16^ All MRI evaluations were performed in a strict research setting, as opposed to clinical, such that accompanying clinical data or other participant information did not influence the evaluations. A subset of NFBC1966 subjects having LBP for 30 days or more over the last 12 months was created to better match the clinical data on which SpineNet was trained. A subset excluding the smallest MC was also evaluated separately. Furthermore, only intervertebral disks from L1-L2 to L5-S1 were included, whereas SpineNet was trained to also evaluate the T12-L1 disk.

For each participant, up to nine slices from the T2-weighted sagittal MRI sequences were passed as a volume to the trained SpineNet model. The model provided output of predictions for Pfirrmann grade 1 to 5 and MC as present or absent at upper and lower vertebral bodies adjacent to the endplate given for each lumbar disk for each participant. We also collected the model outputs as logits and converted these to probabilities for each class.

### SpineNet

SpineNet uses a deep convolutional neural network architecture to provide predictions for a range of lumbar DD features simultaneously using multitask training. At the time of its publication, it was state-of-the-art in the field.^6^ An updated version of SpineNet was made open source in 2022^11^ and the publicly available code and pretrained model weights and parameters were used for this study. This trained model used to evaluate the NFBC1966 MRIs is that which was found to be optimal by the authors based on their own internal validation metrics as described previously^6^ and recently updated.^11^

SpineNet was trained on T2-weighted MRIs from patients recruited as part of the GENODISC consortium project^17^ at multiple centers in the United Kingdom, Hungary, Italy, and Slovenia using a range of different MRI devices and acquisition protocols. All scans were evaluated for multiple DD features including Pfirrmann grades and MC by a single radiologist. The reported intrarater reliability was Cohen’s κ=0.86 and 0.83 for upper and lower marrow changes, and Lin’s concordance correlation coefficient (Lin’s CCC)=0.91 for Pfirrmann grades. The authors used the term “marrow changes” rather than MC because only T2-weighted images were used.

Jamaludin *et al* 6 reported two main summary statistics for interrater reliability between SpineNet and the radiologist on GENODISC data: Lin’s CCC for ordinal data of more than two classes (*i.e.* Pfirrmann grades), and Cohen’s κ for binary data (*i.e.* presence or absence of MC). Lin’s CCC=0.88 and Cohen’s κ=0.63 were reported for SpineNet versus human interrater reliability for Pfirrmann grades and MC, respectively. In the updated version of SpineNet^11^ balanced accuracy scores of 70.9% for Pfirrmann grading and 88.9% and 88.2% were reported for upper and lower MC, respectively. A recent separate external validation of SpineNet included results for Pfirrmann grade classification, with class average accuracy of 55%, weighted Kappa of 0.72, Lin’s CCC of 0.72.^18^ Together, these were the only scores available to serve as the benchmark against which SpineNet and radiologists in Finland could be compared using the previously unseen NFBC1966 data set.

### Agreement and Reliability Metrics

Two groups of agreement and reliability metrics were used to compare the performance of SpineNet against the human raters of NFBC1966 for all participants at all disk levels. Balanced accuracy, Matthew’s correlation coefficient (MCC), receiver operator characteristic curves and their area (AUC), and Brier score loss are commonly used in the DL field to compare the performance of algorithms, whereas metrics such as Cohen κ are more typical in a diagnostic reliability setting. Lin’s CCC was used by the original SpineNet authors as an alternative to Cohen’s κ for the multiclass problem (DD), so we have additionally provided this metric for comparison. In the multiclass setting, the Cohen’s κ formulation is similar to MCC, hence very similar values for MCC and Cohen’s κ for Pfirrmann grades were expected.^19^

Confidence intervals and *P*-values for all agreement and reliability metrics were calculated using a custom nonparametric stratified bootstrap method in Python 3.9 using the SKLearn^20^ and PyCM^21^ packages. McNemar test was used for ascertaining *P*-values for the MC confusion matrix, and in a one versus all approach for the multiclass DD confusion matrix using McNemar-Bowker’s test. A *post hoc* power calculation was carried out to examine the appropriateness of the sample size for calculating Cohen’s κ. Further details are outlined in the Supplemental Materials, Supplemental Digital Content 1, http://links.lww.com/BRS/B981.

GENODISC data represents patients presenting for “secondary care” of LBP, and therefore a clinical population, whereas NFBC participants represent a population cohort for which only a portion suffered LBP. To take this into consideration, agreement and reliability were calculated for both Pfirrmann grades and MC on a subset of participants who suffered LBP on 30 days or more in the last year (prolonged LBP subset).

Because of the heterogeneity of MC evaluation,^22^ agreement and reliability of MC were analyzed including (1) all manually evaluated MC and (2) excluding manually evaluated axially small MC with only one affected axial zone.^16^

## RESULTS

**Figure 2:**
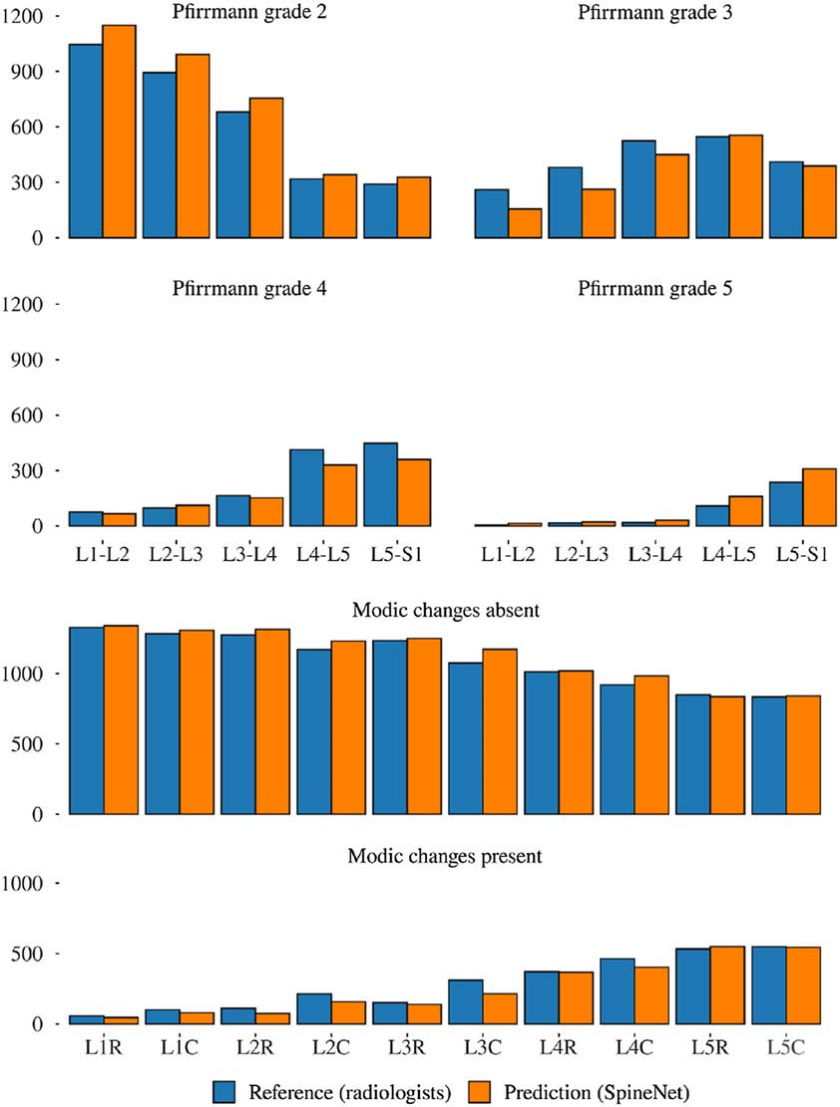
Distribution of reference (radiologist) and predicted (SpineNet) Pfirrmann grades and Modic changes across spinal levels. L1R: rostral to L1-L2 disk; L1C: caudal to L1-L2 disk, etc.

In total, 6655 disks from 1331 participants were analyzed using SpineNet. The distribution across spinal levels for each reference and predicted grade for DD and MC are shown in Figure 2.

Balanced accuracy for Pfirrmann grades was 78% (77%–79%), macroaveraged and microaveraged AUC were 0.94 and 0.95, respectively, whereas Brier score loss was 0.20. Interrater reliability for Pfirrmann grading on NFBC1966 data was Lin’s CCC=0.86 (0.85–0.87) and Cohen’s κ=0.68 (0.67–0.69). In the NFBC1966 prolonged LBP subset, Lin’s CCC was unchanged. In total, 20.83% of disks were rated differently by SpineNet compared with the human raters, but only 0.85% of disks had a difference in grade >1.

**Figure 3:**
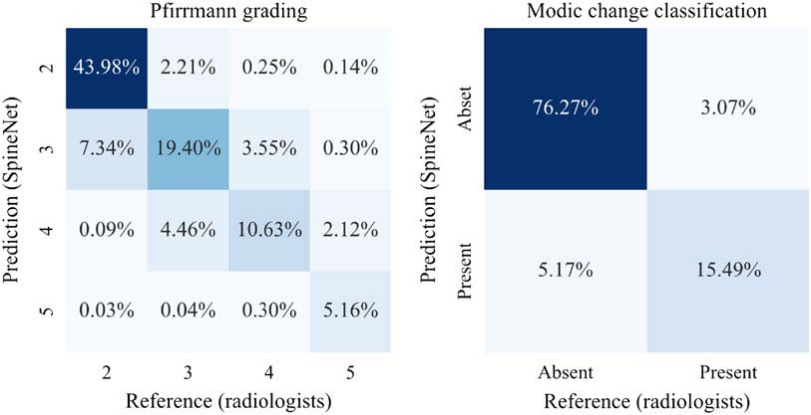
Confusion matrices showing percentages for Pfirrmann grades and Modic changes.

Balanced accuracy for MC was 86% (85%–86%), AUC was 0.95, and Brier score loss was 0.21. Interrater reliability for MC detection was κ=0.74 (0.72–0.75). In the subset with prolonged LBP and where small MC were excluded (n=319), this metric was almost unchanged at κ=0.76 (0.73–0.79). In total 8.24% of MC were classified differently by SpineNet compared with the human raters. These results are summarized in Table 2 and Figure 3, with expanded results available in the supplementary material, Supplemental Digital Content 1, http://links.lww.com/BRS/B981.

**TABLE 2:** Primary Evaluation Metrics With 95% CI

Metric	Pfirrmann grades (%)	Modic changes (%)
Accuracy	79 (78–80)	92 (91–92)
Balanced accuracy	78 (77–79)	86 (85–86)
Macroaveraged AUC	0.95 (0.95–0.95)	—
Microaveraged AUC	0.94 (0.94–0.94)	—
AUC	—	0.95 (0.95–0.95)
Brier score loss	0.20 (0.20–0.20)	0.21 (0.21–0.21)
Sensitivity	79 (78–80)	89 (88–91)
Specificity	93 (93–93)	96 (96–96)
Matthew’s CC	0.68 (0.67–0.70)	0.74 (0.73–0.75)
Cohen’s κ	0.68 (0.67–0.69)	0.74 (0.72–0.75)
Lin’s CCC	0.86 (0.85–0.87)	—
Gwet’s AC1	0.73 (0.72–0.74)	0.88 (0.87–0.89)

AUC indicates area under receiver operator characteristic curve; Gwet’s AC1, Gwet’s agreement coefficient; Lin’s CCC, Lin concordance correlation coefficient; MCC, Matthew’s correlation coefficient.

**Figure 4:**
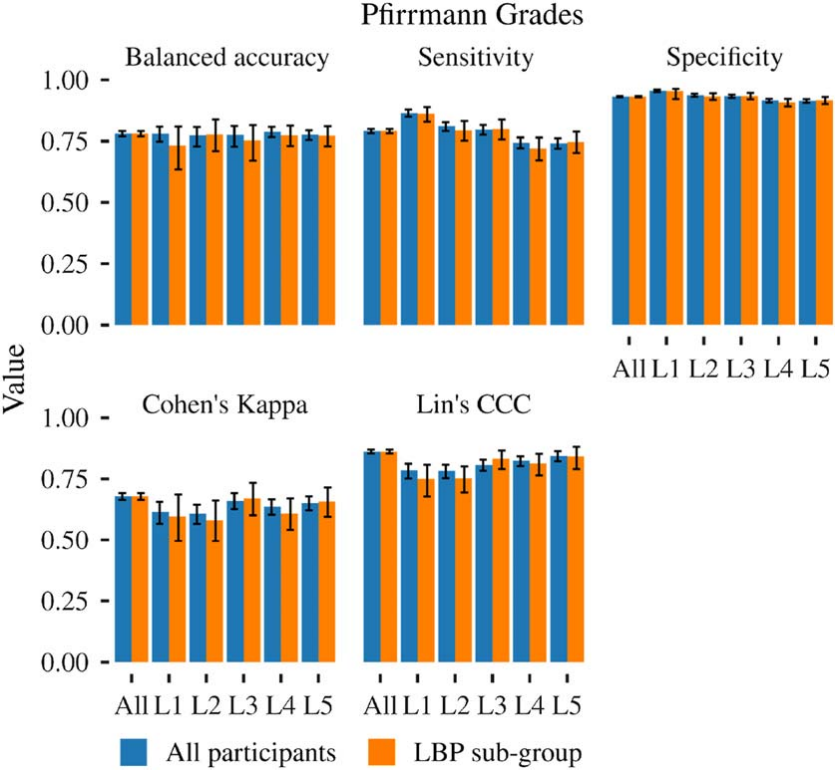
Selected agreement and reliability metrics for Pfirrmann grade predictions at all levels combined and by individual spinal level with error bars showing 95% CIs. LBP subgroup indicates a subset of NFBC subjects having low back pain for 30 days or more over the last 12 months; Lin CCC, Lin concordance correlation coefficient.

**Figure 5:**
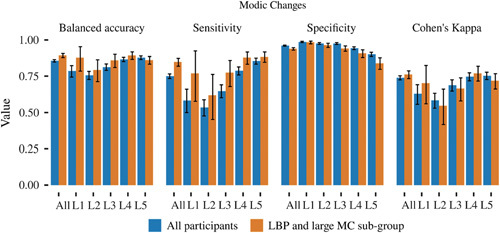
Selected agreement and reliability metrics for Modic change predictions at all levels combined and by individual spinal level with error bars showing 95% CIs. LBP and large MC subgroup: a subset of Northern Finland Birth Cohort subjects having low back pain for 30 days or more over the last 12 months and where the smallest Modic changes in the reference data were ignored (coded as Modic change absent). LBP indicates low back pain; MC, Modic changes.

These results were similar per disk level for the same subsets (Figures 4, 5), although the low prevalence of DD and MC at L1-L3 made the metric estimation less significant at those levels. For all metrics across disk levels, no significant differences were found for the subsets that were aligned more closely with SpineNet training data.

Confusion matrix *P*-values were calculated as *P*<0.0001 for both Pfirrmann grades and MC. A post hoc power calculation indicated that 296 disks were needed for sufficient power for MC and 341 disks for DD, using Cohen’s κ=0.40 as the null hypothesis value.

## DISCUSSION

In this study, SpineNet demonstrated good performance on new data for lumbar DD MRI grading despite the multiple challenges facing the generalizability of a DL model in this context. Interrater reliability between SpineNet and expert human raters for Pfirrmann grades was similar to that reported for the first version of SpineNet, with Lin’s CCC=0.86 versus 0.88, while interrater reliability for MC was increased, with Cohen’s κ=0.74 *versus* κ=0.63. This is clearly a reflection of the developments made to the model between SpineNet versions 1^6^ and 2.^11^ In addition, balanced accuracy (78% and 86% for Pfirrmann grade and MC, respectively) is in line with that reported for version 2 trained on GENODISC data (70% and 89% for Pfirrmann grade and MC, respectively), which provides further evidence that the model is robust in this new setting. Attempting to more closely match the MRI data SpineNet was trained on by subsetting for LBP and MC size did not significantly affect the results for either Pfirrmann grades (Figure 4) or MC (Figure 5). The low prevalence of features at the upper lumbar levels, especially L1-L2 and L2-L3, somewhat limit the significance of the results at these segments due to the large class imbalances.

External validation is essential for the development of any automated medical image classification or diagnostic tool. In LBP research, image classification requires robust tools that can handle data from diverse sources in a consistent manner. External validation is also crucial if DL tools are to transition to clinical environments, where algorithm-informed decisions could significantly impact the care of people suffering LBP. However, external validation can be challenging and time consuming, and it is not always seen as a priority in DL research.^9^,^23^ There is no standardized approach to evaluation that allows easy comparisons between models, and inconsistencies in the use of terminology around model evaluation complicates the issue.^7^,^24^ These difficulties and general lack of robust external validation are some of the key factors that are likely stymieing the development and application of impactful artificial intelligence in spine imaging.^10^

The authors of SpineNet deemed it essential that their system be testable and have made it available as an online demo such that images can be uploaded and the results reported (http://zeus.robots.ox.ac.uk/spinenet2/), in addition to the source code made available on GitHub. This openness to external testing of a trained DL model in the field of medical image classification and diagnostics sets a noteworthy precedent. Among published DL models for grading lumbar DD MRI features, few have been externally validation on geographically distinct data.

The results show comparable performance on the main metrics for reliability reported by Jamaludin *et al* 6 and Windsor. *et al*.^11^ We previously validated a proprietary version of SpineNet, where the reliability was significantly lower (Cohen’s κ=0.51 and 0.55 for Pfirrmann grading and MC, respectively), and it is clear that significant improvements have been delivered with the publication of this open-source version. As such, SpineNet has performed well considering multiple challenges in this setting. Although the diverse sources for the training sample used by SpineNet may strengthen the model’s generalizability, the fact that a single rater was used for all scans that the algorithm trained on can be considered a weakness. Furthermore, the radiologists grading the NFBC1966 MRIs did not differentiate between Pfirrmann grade 1 and 2, considering both to be normal. This is an example of the idiosyncrasies encountered when working with geographically separated samples. Less measurable but equally significant differences are likely present in the qualitative interpretation of the images,^25^ along with differences in MRI acquisition protocols and equipment contributing to concept and data drift. These factors resulting from domain shift between training and external validation data sets may impact the generalizability of SpineNet, but it seems that on NFBC1966 data this has not been a significant issue. The SpineNet training data were not available for this study, precluding a detailed assessment of the extent to which domain shift or data set similarity may have impacted SpineNet’s reliability.

**Figure 6:**
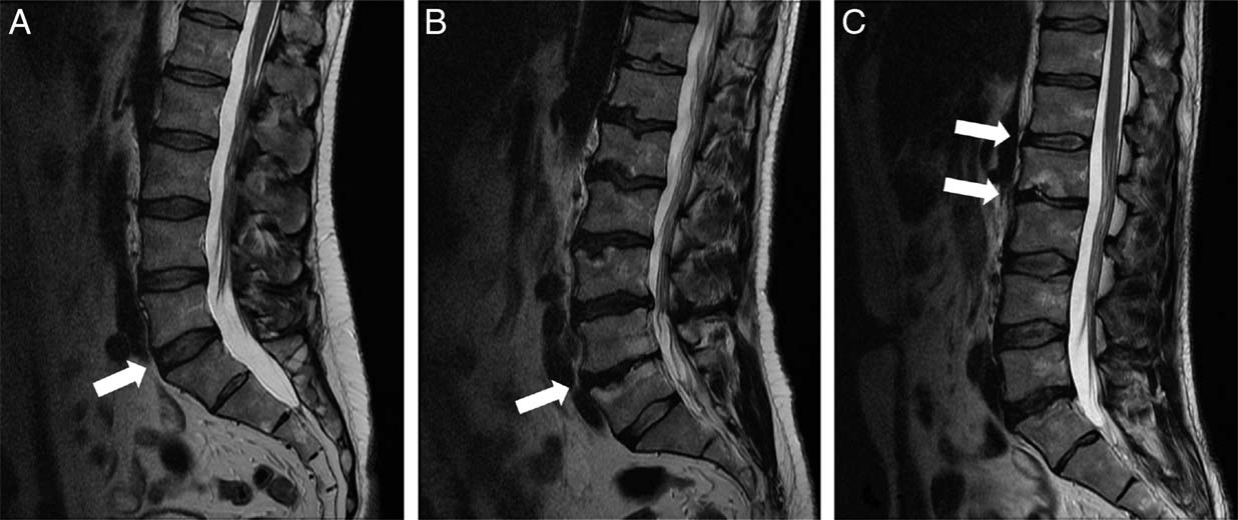
Lumbar T2 magnetic resonance imaging mid-sagittal slices showing cases of disagreement on Pfirrmann grade between SpineNet and Finnish radiologists. A, L5-S1 intervertebral disk coded as Pfirrmann grade 5 by SpineNet but 2 by radiologists. B, L5-S1 intervertebral disk coded as Pfirrmann grade 2 by SpineNet but 5 by radiologists. C, L1-L2 intervertebral disk coded as Pfirrmann grade 5 by SpineNet but 2 by radiologists, L2-L3 intervertebral disk coded as Pfirrmann grade 2 by SpineNet but 5 by radiologists.

**Figure 7:**
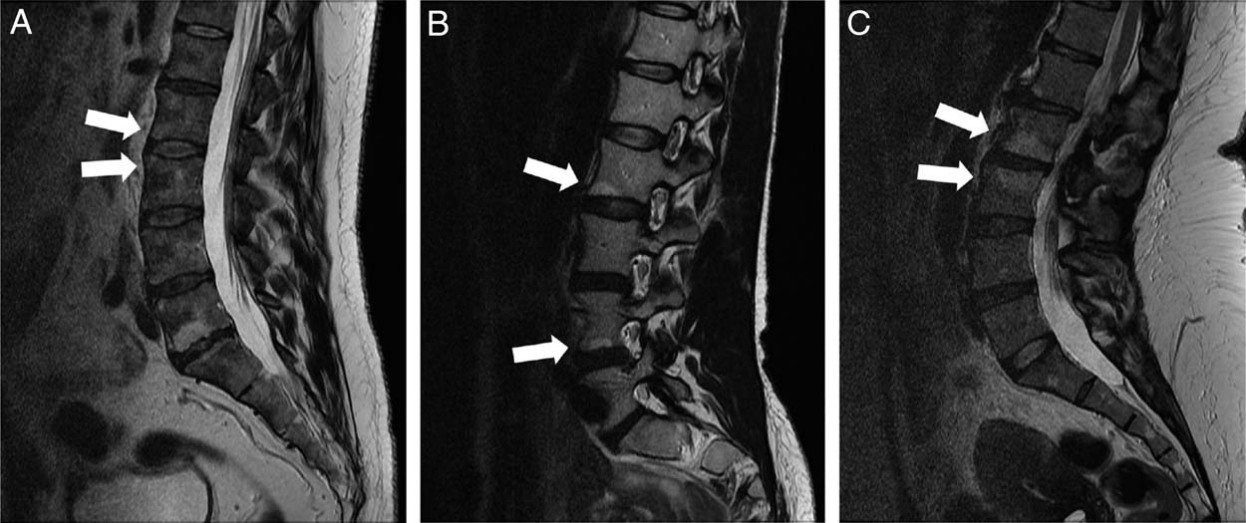
Lumbar T2 magnetic resonance imaging slices showing cases of disagreement on Modic change classification between SpineNet and Finnish radiologists. A, Mid-sagittal slice of L2-L3 intervertebral disk with upper and lower end plates coded as having Modic changes present by SpineNet but coded as having no Modic changes by radiologists. B, Lateral sagittal slice of L2-L3 and L4-L5 intervertebral disks with upper end plates coded as having no Modic changes by SpineNet but coded as having Modic changes by radiologists. C, Mid-sagittal slice of L2-L3 intervertebral disk upper and low end plates coded as having no Modic changes by SpineNet but coded as having Modic changes by radiologists.

Examples of poor predictions by SpineNet are shown in Figure 6 for Pfirrmann grading and in Figure 7 for MC. It is clear from these examples that the presence of lumbosacral transitional vertebra is a major challenge to the model. This anatomic variation is relatively prevalent with some form of lumbosacral transitional vertebra found in up to 29% of participants in a Finnish population cohort study.^26^ Such transitional vertebrae can alter the numbering of vertebral levels and a mislabeling of L5 as S1 or vice versa will cause estimates for all levels to be shifted cranially or caudally, potentially resulting in disagreement at every level in that participant. Despite these various factors with the potential to undermine the performance of SpineNet on NFBC1966 data, the results are inside the range of what has been previously reported for human versus human interrater reliability, at least for Pfirrmann grades (Cohen’s κ=0.59–0.81, Table 3). These results are also similar to those for multiple other LBP physical examinations and a wide range of medical tests.^5^ This reflects the irreducible error inherent in human assessment of medical data, something that has long been recognized.^28^ Particularly in LBP research, this has likely undermined the ability of lumbar spine MRI to effectively stratify patients based on MRI or contribute reliable imaging phenotypes.^29^

**TABLE 3:** Comparison of Results of this Study to other Examples for Pfirrmann Grading Intrarater and Interrater Reliability

	Source	Cohen’s κ	Lin’s CCC	Gwet’s AC
Human intrarater	Pfirrmann *et al* 4	0.84–0.90	—	—
	Carrino *et al* 5	0.74	—	—
	Doktor *et al* 27	0.68	—	0.90
	Niemeyer *et al* 7	0.87	0.92	—
	Jamaludin *et al* 6	—	0.91	—
Human interrater	Pfirrmann *et al* 4	0.69–0.81	—	—
	Carrino *et al* 5	0.66	—	—
	Niemeyer *et al* 7	0.59	0.62	—
	Mertimo *et al* 15	0.39–0.79	—	—
Human *vs.* DL algorithm interrater	Niemeyer *et al* 7	0.92	0.94	—
	Jamaludin *et al* 6	—	0.88	—
	This study	0.68	0.86	0.73

DL indicates deep learning; Gwet’s AC, Gwet’s agreement coefficient; Lin’s CCC, Lin’s concordance correlation coefficient.

In this study, SpineNet has been benchmarked against expert human raters in the research setting. This is important because in the clinical setting the evaluator is appraised of the clinical situation, which results in both intended and unintended biases applied to the resulting evaluations.^30^ The strict nature of the research setting, the ability to amalgamate consensus evaluations, and the separation of biometric and clinical information from the image being interpreted strengthen the conclusions of this study.^9^

The results of this external validation illustrate some of the challenges in testing DL models with previously unseen data. This study is limited by its retrospective design^31^,^32^ and by not making a direct comparison between the distributions of the data used to train SpineNet and to externally validate it. Doing so would have allowed us to estimate the impact of domain shift on the results and is recommended for external validation studies in artificial intelligence when the data are available.^32^ We created subsamples of the data set as an attempt to more closely match the clinical cohort used to train SpineNet, but this did not add further insight.

DL tools trained to replicate expert radiologists’ grading of spine MRI for DD features are unlikely to solve fundamental issues with existing DD and MC imaging phenotypes that undermine LBP association studies. However, they set the stage for future approaches and already offer novel ways of rapidly evaluating large spine MRI data sets as demonstrated by Jamaludin *et al* 27,33 Future work should endeavor to move beyond qualitative spine MRI evaluation and leverage DL for fully data-driven imaging phenotypes and disease prognostication. In this study, SpineNet has demonstrated good performance on a geographically and temporally distinct data set, which is within the bounds of what has been reported in the literature to date. As DL models applied to spine imaging phenotypes develop, testing their robustness through external validation methods such as those used in this study will become increasingly important.

Key Points

- Automated interpretation of lumbar spine MRI using DL could lead to discovery of novel DD imaging phenotypes that are more objective and standardized.
- Development of DL models to achieve this necessitates robust external validation for translation to research and clinical practice.
- In this study, we externally validated SpineNet, a state-of-the-art MRI DD classification tool, which achieved reliability of Cohen’s κ=0.68 for Pfirrmann grading and κ=0.74 for Modic change identification.

## Supplementary Material

SUPPLEMENTARY MATERIAL
