# Supplementary material for: External Validation of SpineNet, an Open-Source Deep Learning Model for Grading Lumbar Disk Degeneration MRI Features, Using the Northern Finland Birth Cohort 1966
Source: Spine (Phila Pa 1976). 2022 Dec 30;48(7):484–91. doi: 10.1097/BRS.0000000000004572 (PMC9990601; doi:10.1097/BRS.0000000000004572)
Supplement: SUPPLEMENTARY MATERIAL [file brs-48-484-s001.pdf]

Supplementary Materials

Supplementary Methods

Confidence intervals and p-values for all metrics were calculated using nonparametric bootstrapping methods using a custom Python script. P-values for agreement and reliability metrics can be calculated with a one-tailed test and null hypothesis value of zero. However, metrics will usually be better than that expected by chance so specifying a null hypothesis of zero is not particularly meaningful [1]. For metrics such as Cohen's κ, MCC, Lin's CCC, and Gwet's AC, we used a "minimum acceptable level of agreement" based on qualitative interpretations of those metrics (e.g., Cohen's k and MCC 0.40). For Accuracy, balanced accuracy, sensitivity, and specificity we used a naïve classifier (no information rate in this case) as the null hypothesis (see **Supplementary Tables 1** and **2**).

Supplementary Results

Further characteristics of the dataset used in this study are shown in **Supplementary Figure 1**, with the distribution of MC width shown across spinal levels. The low prevalence of MC at upper lumbar levels likely contributes to the low sensitivity and high specificity of SpineNet compared to radiologists at these levels (**Supplementary Figure 2** and **3**).

Probability distributions were examined, and the expected calibration error calculated as 7.45 for Pfirrmann grading and 3.38 for Modic changes. This information is summarized graphically in **Supplementary Figure 4**.

Receiver operator characteristic curves (ROC) and areas (AUC) have been calculated to give further insight into model performance (**Supplementary Figure 5** and **6**). However, for the multiclass setting of Pfirrmann grading the ROC and AUC are less informative as, unlike in the binary classification setting,

they are sensitive to class imbalance. Nonetheless we present the one vs all approach for each Pfirrmann grade along with the macro- and micro-averaged ROC and AUC.

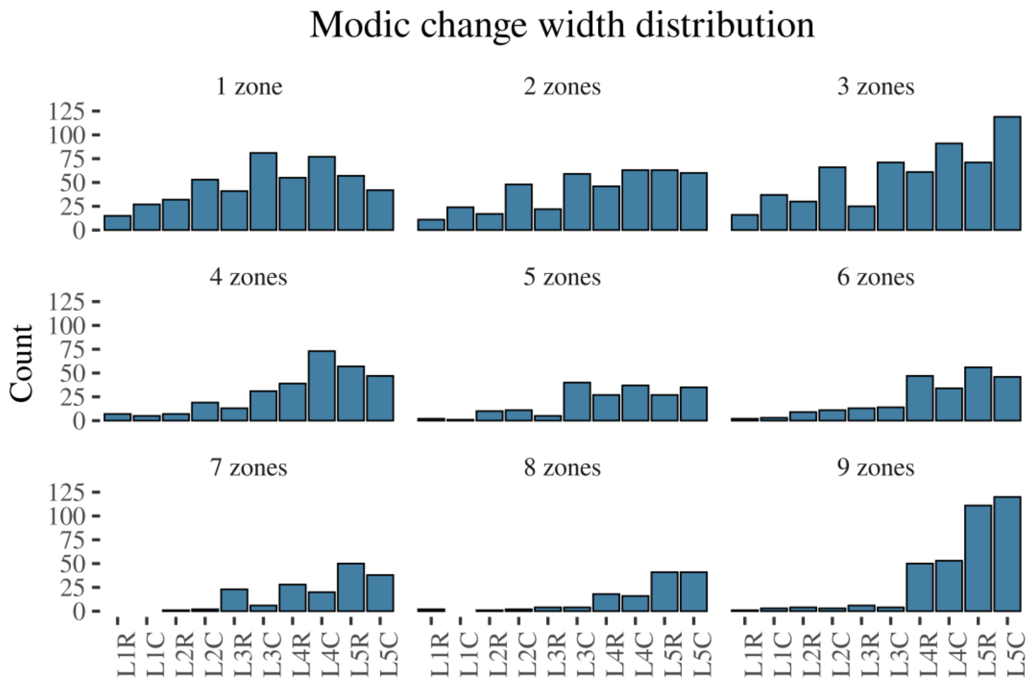

Supplementary Figure 1 Distribution of Modic change widths across spinal levels. 1 represents Modic changess present in a single zone of the vertebral endplate, while 9 represents Modic changes in all 9 zones of the endplate, based on the criteria defined by Määttä et al, 2015 (17). L1R: rostral to L1-L2 disc; L1C: caudal to L1-L2 disc; etc.

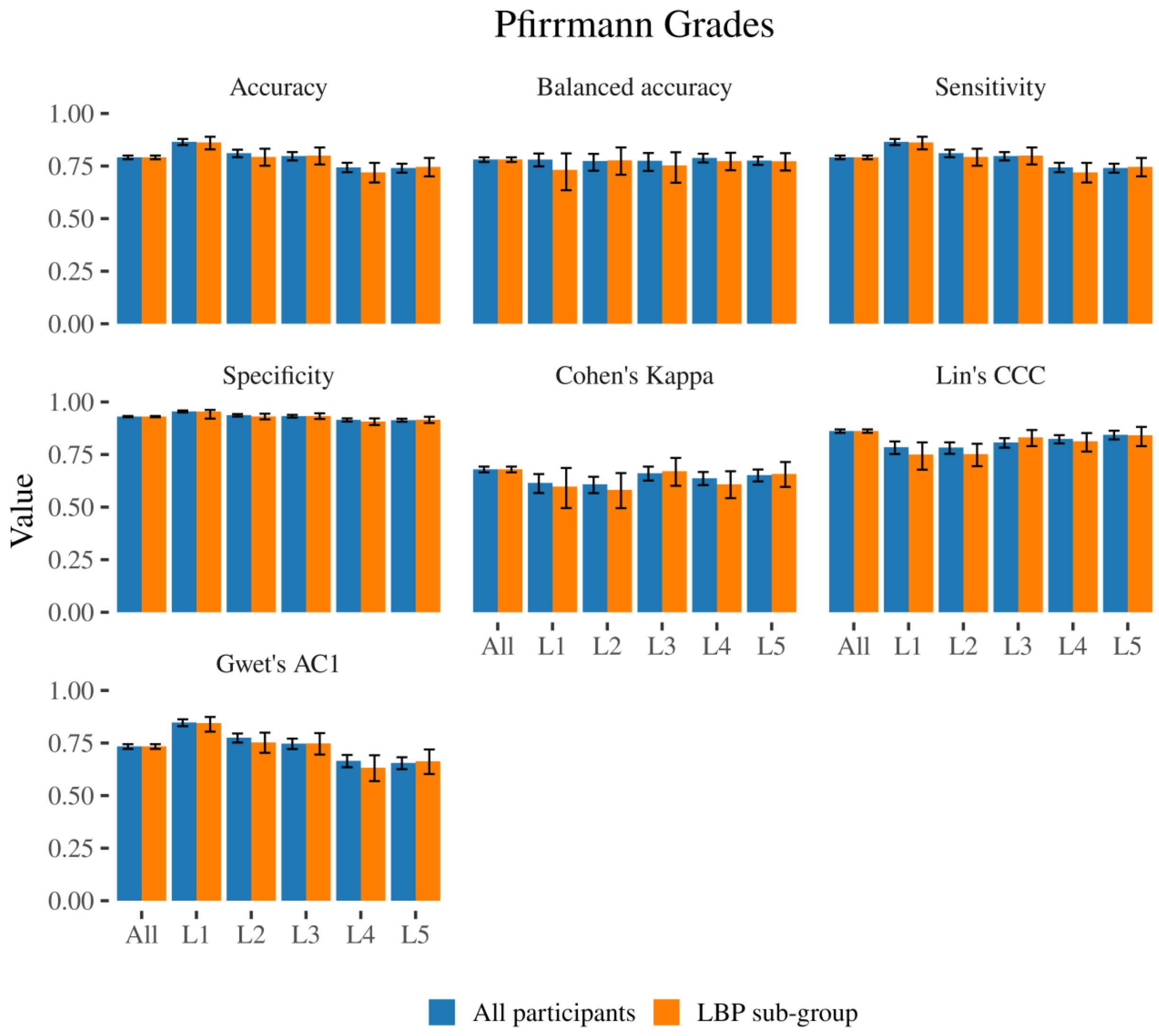

Supplementary Figure 2 All calculated agreement and reliability metrics for Pfirrmann grade predictions at all levels combined and by individual spinal level with error bars showing 95% confidence intervals. LBP and large MC sub-group: a subset of Northern Finland Birth Cohort subjects having low back pain for 30 days or more over the last 12 months; Lin's CCC: Lin's concordance correlation coefficient.

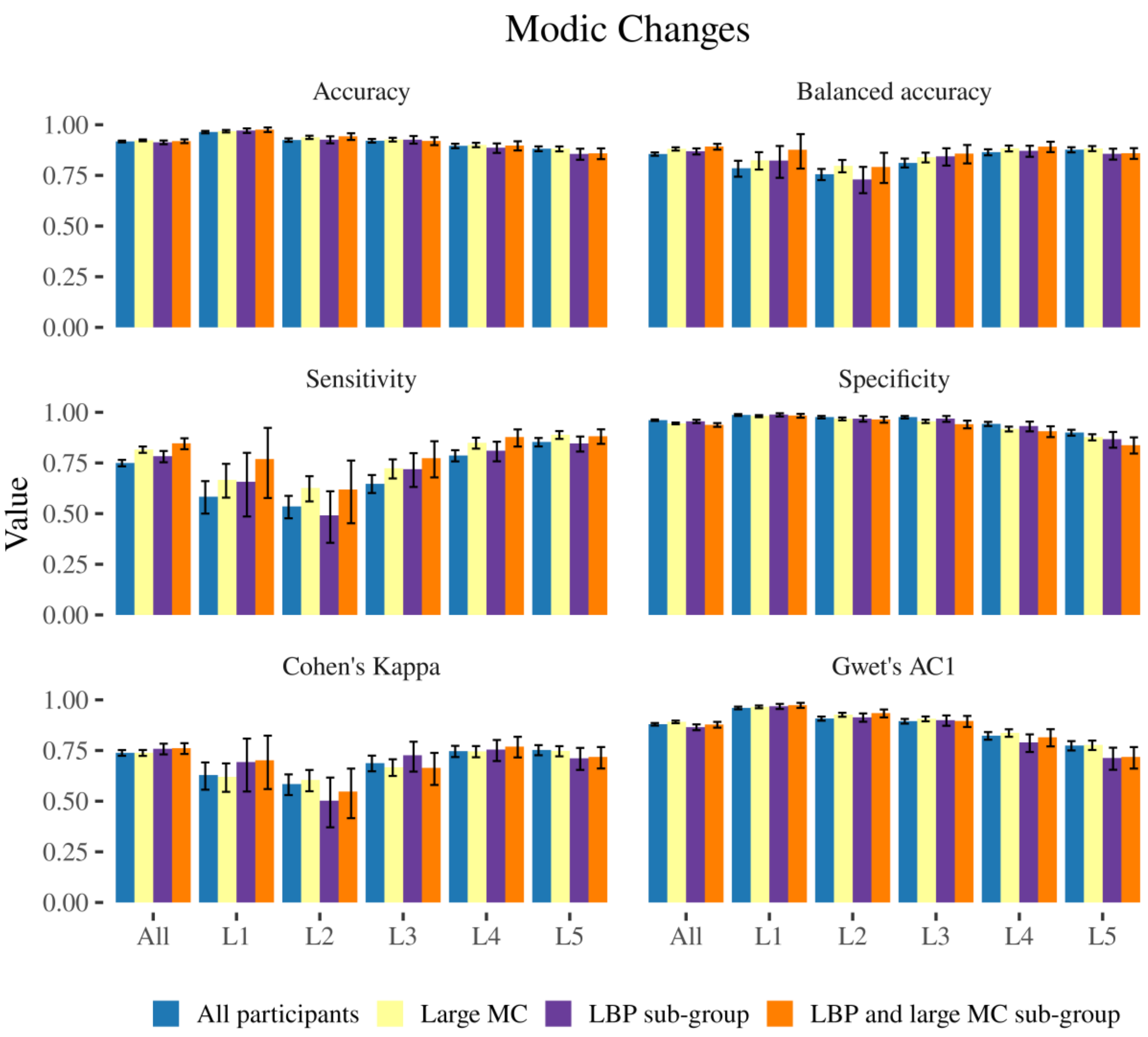

Supplementary Figure 3 All calculated agreement and reliability metrics for Modic change predictions at all levels combined and by individual spinal level with error bars showing 95% confidence intervals. LBP and large MC sub-group: a subset of Northern Finland Birth Cohort subjects having low back pain for 30 days or more over the last 12 months and where the smallest Modic changes in the reference data were ignored (coded as MC absent); LBP: low back pain; MC: Modic change; Gwet's AC1: Gwet's agreement coefficient 1.

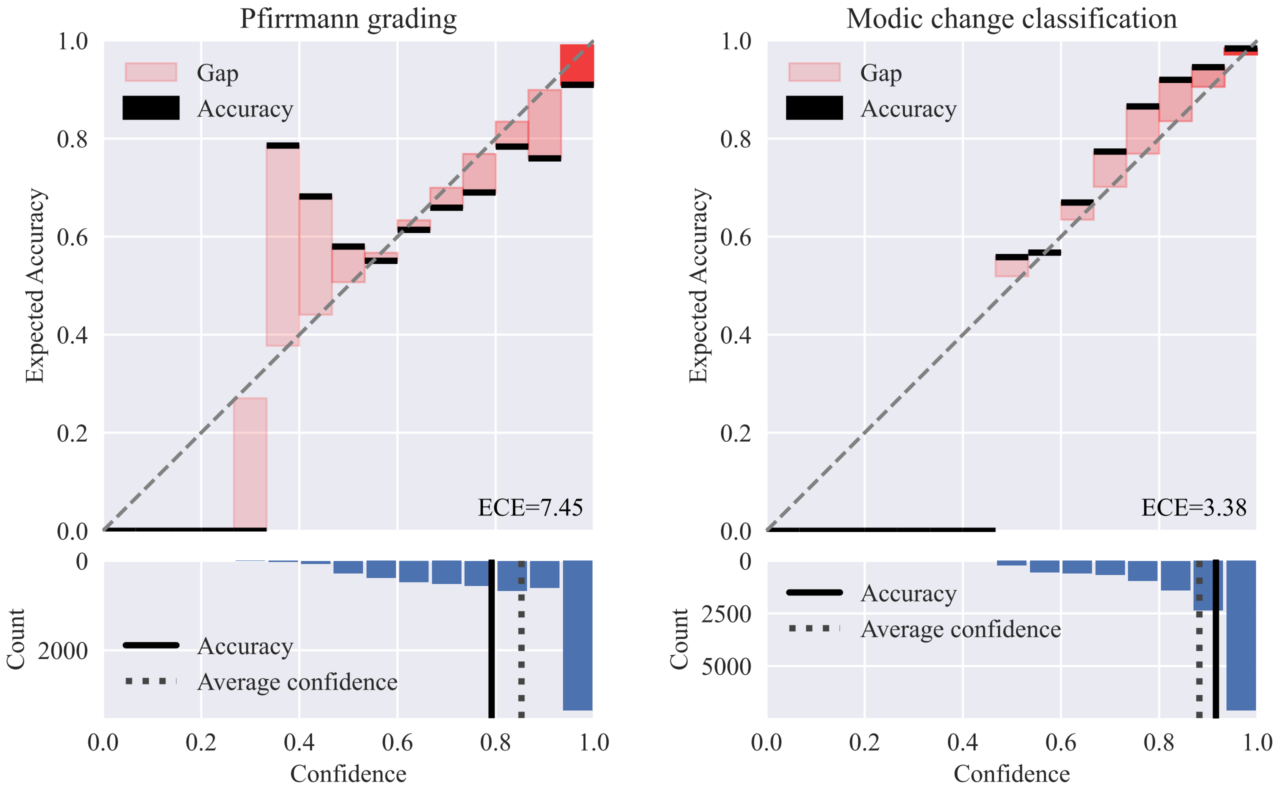

Supplementary Figure 4 Reliability diagrams (top) and confidence histograms (bottom). ECE: expected calibration error.

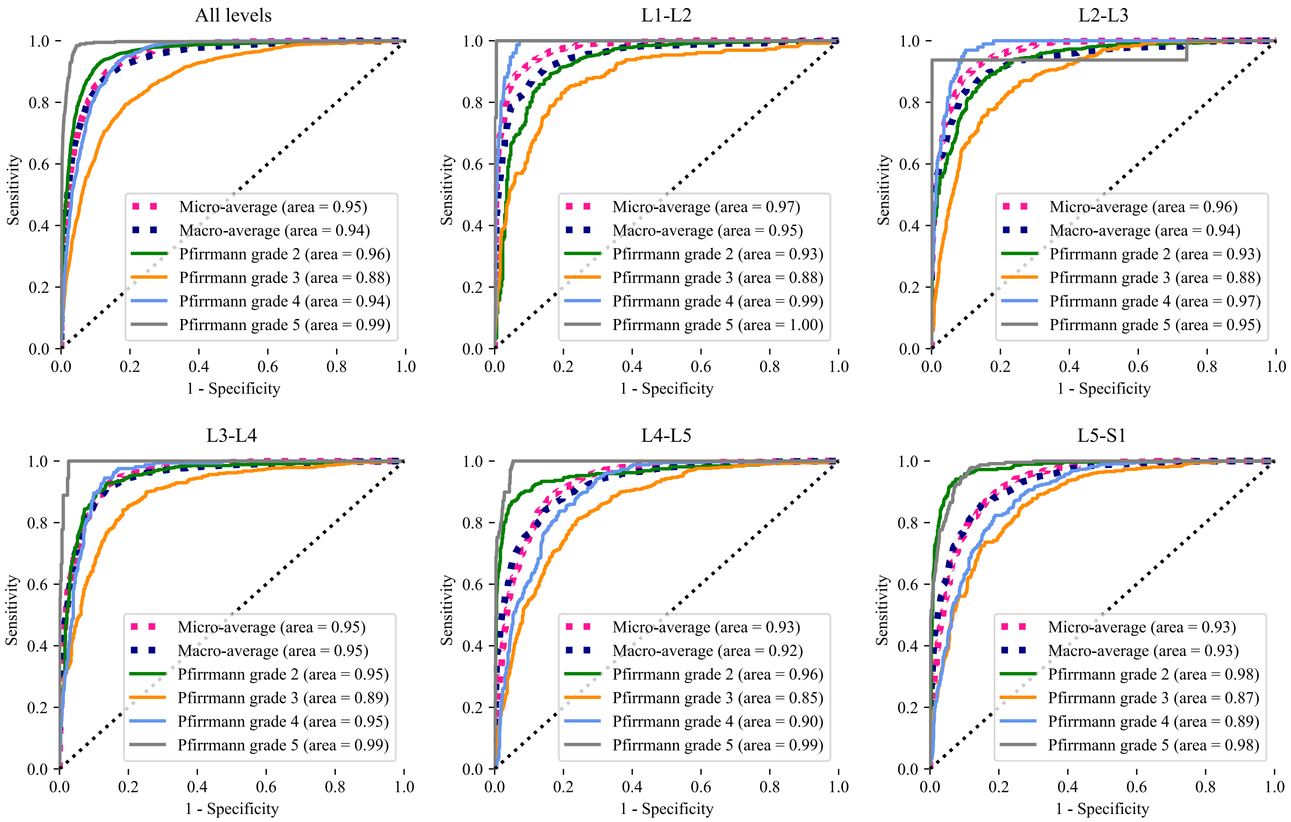

Supplementary Figure 5 ROC and AUC for each class and macro- and micro-averaged by vertebral level.

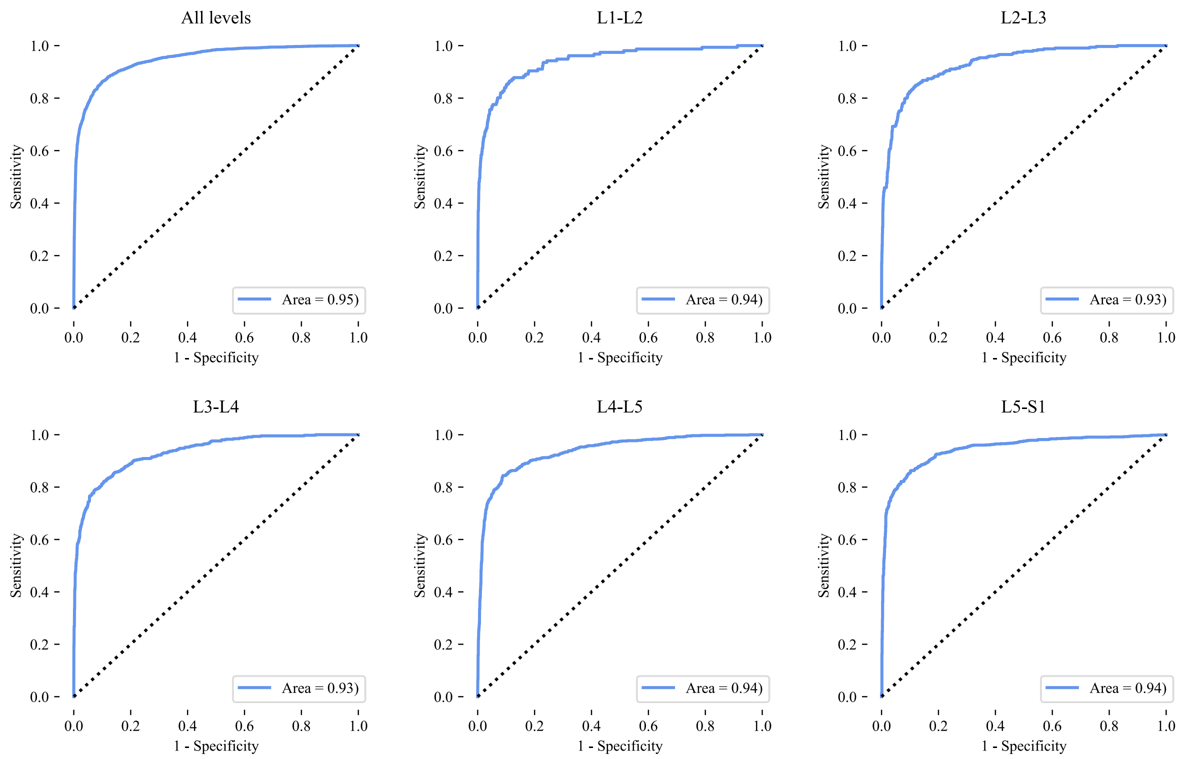

Supplementary Figure 6 ROC curves and AUC for Modic changes by vertebral level.

Supplementary Table 1 Brier score loss by level.

Level	Pfirrmann grading Brier score loss	Modic change classification Brier score loss
All levels	0.20	0.21
L1-L2	0.13	0.06
L2-L3	0.17	0.12
L3-L4	0.20	0.17
L4-L5	0.24	0.30
L5-S1	0.24	0.39

Supplementary Table 2 All results, confidence intervals, null hypotheses, and p-values for all metrics across spinal levels and subgroups for Pfirrmann grades. CI: confidence interval; H0: null hypothesis; Lin's CCC: Lin's concordance correlation coefficient; Gwet's AC1: Gwet's agreement coefficient; MCC: Matthew's correlation coefficient.

Metric	Group	Value	Lower CI	Upper CI	H0	p-value
Accuracy	All	0.79	0.78	0.80	0.47	<0.0001
	LBP	0.79	0.78	0.80	0.47	<0.0001
Balanced accuracy	All	0.78	0.77	0.79	0.25	<0.0001
	LBP	0.78	0.77	0.79	0.25	<0.0001
Cohen's κ	All	0.68	0.67	0.69	0.40	<0.0001
	LBP	0.68	0.67	0.69	0.40	<0.0001
Lin's CCC	All	0.86	0.85	0.87	0.40	<0.0001
	LBP	0.86	0.85	0.87	0.40	<0.0001
Gwet's AC	All	0.73	0.72	0.74	0.40	<0.0001
	LBP	0.73	0.72	0.74	0.40	<0.0001
MCC	All	0.68	0.67	0.70	0.40	<0.0001
	LBP	0.68	0.67	0.70	0.40	<0.0001
Sensitivity	All	0.79	0.78	0.80	0.50	<0.0001
	LBP	0.79	0.78	0.80	0.50	<0.0001
Specificity	All	0.93	0.93	0.93	0.50	<0.0001
	LBP	0.93	0.93	0.93	0.50	<0.0001

Supplementary Table 3 All results, confidence intervals, null hypotheses, and p-values for all metrics across spinal levels and subgroups for Modic changes. CI: confidence interval; H0: null hypothesis; Gwet's AC1: Gwet's agreement coefficient; MCC: Matthew's correlation coefficient.

Metric	Group	Value	Lower CI	Upper CI	H0	p-value
Accuracy	All	0.92	0.91	0.92	0.79	<0.0001
	LBP	0.91	0.90	0.92	0.76	<0.0001
	MC size	0.92	0.92	0.93	0.83	<0.0001
	MC size and LBP	0.92	0.91	0.93	0.79	<0.0001
Balanced accuracy	All	0.86	0.85	0.86	0.50	<0.0001
	LBP	0.87	0.85	0.88	0.50	<0.0001
	MC size	0.88	0.87	0.89	0.50	<0.0001
	MC size and LBP	0.89	0.91	0.92	0.50	<0.0001
Cohen's κ	All	0.74	0.72	0.75	0.40	<0.0001
	LBP	0.76	0.73	0.78	0.40	<0.0001
	MC size	0.74	0.72	0.75	0.40	<0.0001
	MC size and LBP	0.76	0.73	0.79	0.40	<0.0001
MCC	All	0.74	0.73	0.75	0.40	<0.0001
	LBP	0.76	0.73	0.78	0.40	<0.0001
	MC size	0.74	0.72	0.75	0.40	<0.0001
	MC size and LBP	0.76	0.73	0.79	0.40	<0.0001
Gwet's AC	All	0.88	0.87	0.89	0.40	<0.0001
	LBP	0.87	0.85	0.88	0.40	<0.0001
	MC size	0.89	0.88	0.90	0.40	<0.0001
	MC size and LBP	0.88	0.86	0.89	0.40	<0.0001
Sensitivity	All	0.89	0.88	0.91	0.50	<0.0001
	LBP	0.75	0.73	0.77	0.50	<0.0001
	MC size	0.78	0.75	0.81	0.50	<0.0001
	MC size and LBP	0.82	0.80	0.83	0.50	<0.0001
Specificity	All	0.96	0.96	0.96	0.50	<0.0001
	LBP	0.96	0.95	0.96	0.50	<0.0001
	MC size	0.95	0.94	0.95	0.50	<0.0001
	MC size and LBP	0.94	0.93	0.95	0.50	<0.0001

Supplementary References

1. Sim J, Wright CC. The Kappa Statistic in Reliability Studies: Use, Interpretation, and Sample Size Requirements. Phys Ther. 2005;85:257–68.
